# Epidemiological pathology of Tau in the ageing brain: application of staging for neuropil threads (BrainNet Europe protocol) to the MRC cognitive function and ageing brain study

**DOI:** 10.1186/s40478-016-0275-x

**Published:** 2016-02-08

**Authors:** Stephen B. Wharton, Thais Minett, David Drew, Gillian Forster, Fiona Matthews, Carol Brayne, Paul G. Ince, on behalf of the MRC Cognitive Function and Ageing Neuropathology Study Group

**Affiliations:** Sheffield Institute for Translational Neuroscience, University of Sheffield, 385A Glossop Road, Sheffield, S10 2HQ UK; Department of Radiology, University of Cambridge, Cambridge, UK; Institute of Public Health, University of Cambridge, Cambridge, UK; MRC Biostatistics Unit, University of Cambridge, Cambridge, UK

**Keywords:** Alzheimer’s disease, Brain ageing, Tauopathy, Neuropil threads, Staging schemes, Thorn-shaped astrocytes, Braak Stage, BrainNet Europe

## Abstract

**Introduction:**

Deposition of abnormally phosphorylated tau (phospho-tau) occurs in Alzheimer’sdisease but also with brain ageing. The Braak staging scheme focused on neurofibrillary tangles, butabundant p-tau is also present in neuropil threads, and a recent scheme has been proposed by theBrainNet Europe consortium for staging tau pathology based on neuropil threads. We determined therelationship of threads to tangles, and the value of staging for threads in an unselected population-representative ageing brain cohort. We also determined the prevalence of astroglial tau pathologies, and their relationship to neuronal tau. Phospho-tau pathology was determined by immunohistochemistry (AT8 antibody) in the MRC-CFAS neuropathology cohort. Neuropil threads were staged using the BrainNet Europe protocol for tau pathology, and compared with Braak tangle stages. Astroglial tau pathology was assessed in neo-cortical, mesial temporal and subcortical areas.

**Results:**

Cases conformed well to the hierarchical neuropil threads staging of the BrainNet Europe protocol and correlated strongly with Braak staging (*r*=0.84, *p* < 0.001). Based on the areas under the receiver operator curves (AUC), incorporating either threads or tangle staging significantly improved dementia case identification to a similar degree over age alone (Braak stage *X*^2^(1)=10.1, *p*=0.002; BNE stage *X*^2^(1)=9.7, *p*=0.002). Thorn-shaped astrocytes, present in 40 % of cases, were most common in mesial temporal lobe, then brainstem, and were associated with subpial tau-positive neurites (mesial temporal: *X*^2^(1)=61.3, *p* < 0.001; brainstem: *X*^2^(1)=47.9, *p* < 0.001). Adding thorn astrocytes did not improve dementia prediction (AUC: *X*^2^(1)=0.77, *p*=0.381), but there was a weak relationship between numbers of areas involved and staging for threads or tangles (*r*=0.17, *p*=0.023). Neuropil threads develop hierarchically in parallel with neurofibrillary tangles.

**Conclusions:**

The BrainNet Europe staging protocol is straightforward to apply, and offers similar predictive value for dementia to Braak tangle staging. Astroglial tauopathy, particularly thorn shaped astrocyte formation, does not relate to dementia status, but the association with phospho-tau neurites may suggest a pathogenic relationship to neuronal tau pathology.

## Introduction

Deposition of abnormal forms of the microtubule-associated protein tau, including phosphorylated forms (p-tau), occurs in a variety of inclusions in neurones and glia, and is of diagnostic utility, in a subset of neurodegenerative disorders referred to collectively as the tauopathies [15]. In Alzheimer’s disease (AD), deposition as neurofibrillary tangles (NFTs), pre-tangles, neuropil threads (NTs), dystrophic neurites in neuritic plaques, and granulovacuolar degeneration, is associated with βA4-amyloid deposition. Identification of mutations of the *MAPT* gene, which encodes tau, in a subset of the frontotemporal lobar degenerations (FTLD-TAU) confirmed the molecular pathogenicity of abnormal tau proteins [9].

However, p-tau deposition is also common in the ageing brain, particularly as AD-type neuropathological lesions, along with AD-type deposits of β-amyloid. Studies in the population-representative Medical Research Council Cognitive Function and Ageing Study have shown overlap in burden of AD pathology in the brains of older people with and without dementia at death, such that thresholds for the prediction and diagnosis of dementia cannot be defined [22, 25]. This is supported by findings in other large unselected or community-based autopsy series showing AD-type lesions in non-demented individuals [3, 10, 12, 14, 18]. The overlap increases in the oldest old such that the relationship of plaques and NFTs to dementia becomes increasingly attenuated [11, 27, 28]. Conversely, a subset of elderly individuals who have only minimal AD pathology are demented, suggesting a role for other factors, such as oxidative damage [31]. This corresponds to cases reported in other series that have an AD-like clinical presentation but with insufficient neuropathology to explain dementia [30]. Neurofibrillary tangle stage is a better correlate with dementia than plaques, but these findings suggest a need to find other drivers of cell dysfunction in the ageing brain and also to better define AD type pathologies at cellular and molecular levels.

NFT pathology in AD, and ageing, progresses in a stereotyped, hierarchical manner and therefore can be staged using the 6-tiered scheme devised by Braak [5]. NFTs may be assessed using either silver impregnation or by immunohistochemistry to abnormally phosphorylated tau protein [4], the latter providing more uniformity in detection and assessment [2]. Much of the tau pathology, however, lies within neuritic processes as neuropil threads. Recently a 6-stage scheme based on the assessment of NTs was devised by the BrainNet Europe consortium [1]. NT pathology progresses anatomically in a similar hierarchy to tangles.

Given the abundance of tau pathology within the neuritic compartment, it is possible that this form of tau pathology has an important influence on cognition. We therefore evaluated the BrainNet Europe neuropil thread staging scheme (BNE NT staging) in the CFAS ageing brain cohort, compared this with Braak NFT staging and assessed the relationship of NTs to dementia. The population-based and unselected nature of the CFAS cohort is well-suited to this study because the cohort allows assessments of the relationships between pathology and dementia in an unbiased way, without the ceiling-floor effects and other biases inherent in case—control cohorts pre-selected by clinical presentation [36]. In addition we examined other age-related forms of p-tau pathology, particularly astroglial tau, with a view to obtaining better prevalence data for age-related tau pathology and evaluating the relationship of these less investigated pathologies to cognition.

## Materials and methods

Tissue was obtained from the neuropathology cohort of the MRC-CFAS [reviewed in [36]] following Research Ethics Committee approval. Two entire sub-cohorts were used to maintain the unbiased population-representative base of the study (Cambridge and Newcastle), totalling 183 cases Neuropathological lesions were previously assessed in this cohort, including AD-neuropathology using the CERAD protocol [23] and Braak NFT stage, based on immunohistochemistry and silver staining. Dementia status at death was based on all information available for each study participant, including algorithmic assessment in life (AGECAT), information from death certification and a Retrospective Informant Interview (RINI) developed by CFAS [20, 24].

NTs were staged using the BNE NT staging protocol [1] in sections immunostained for p-tau using the AT8 antibody, which is commonly used in diagnostic practice and identifies tau phosphorylated at serine 202/threonine 205. Sections from tissue blocks of the following areas were stained: temporal cortex (Brodmann Area 21/22); occipital cortex (BA17/18); posterior hippocampus and adjacent mesial temporal structures; anterior hippocampus and adjacent mesial temporal structures (where available); midbrain; pons; striatum. Briefly, for BNE NT stage, threads were semi–quantified as 0, +, ++ or +++, in comparison with the published reference images, in transentorhinal cortex, entorhinal cortex, occipitotemporal gyus, lateral temporal cortex (BA22/21), occipital association cortex (BA18) and primary visual cortex (BA17) entorhinal and transentorhinal cortex, allowing distinction of 6 stages based on the progressive recruitment of these anatomical areas by neuropil threads of ++ of +++ intensity. The presence of tau-positive neurons (tangles and pre-tangles) was also noted.

In addition to this BrainNet protocol, NTs and NFTs were also noted in subcortical structures (midbrain, pons and striatum). Additional cytopathologies were also noted in all of these cortical and subcortical areas, namely astroglial tau pathologies, particularly thorn-shaped astrocytes (TSA), subpial tau in neuritic process at the brain surface, brainstem subependymal tau processes, argyrophilic grains.

Immunohistochemisty was performed using a standard avidin biotin method combined with diaminobenzidine (Vector Laboratories, Peterborough, UK). Haematoxylin and eosin was used as a counterstain. Phosphorylated tau was detected using the AT8 antibody (1:400, microwave antigen retrieval for 10 mins in trisodium citrate).

### Statistical analysis

Means (standard deviations) are reported. Differences between means were tested using Student *t* test (t) for independent samples. The chi-square test (*X*^2^) was used for comparison of categorical data. Spearman coefficient was calculated to verify the correlation between Braak NFT stage, BNE NT stage and TSA. To verify the influence of age on Braak NFT stage, BNE NT stage and TSA ordinal logistic regression was used with BNE NT stage, Braak NFT stage and TSA as dependent variables and age as independent variable, whereas the relationship of age, Braak NFT stage, BNE NT stage and TSA with dementia was tested using logistic regression with dementia status as dependent variable. Receive operator curve (ROC) regression analyses were performed to verify that adding Braak NFT stage, BNE NT stage or TSA would better discriminate between dementia status than when only age was considered in the model. The chi-square test (*X*^2^) for the equality of areas under the ROC curves (AUC) was used. All tests were 2-tailed. Statistical analyses were performed using statistical package STATA, version 12.

## Results

In total, 183 brains were included in the sample. Among them, 112 (61 %) were women. Mean age of death was 85.9(7.7) years. Dementia at death was present in 106 (58 %) of cases, 69(38 %) did not have dementia and dementia status at death was could not be determined in 8(4 %) participants.

### Neuropil threads

Cases conformed well to the hierarchical sequence of anatomical involvement implicit in the BrainNet Europe and Braak staging protocols, with only two cases (1.1 %) showing minor deviation. One case showed ++ neuropil thread staining in the occipital region, but only + in temporal, thus bypassing this area in terms of stage progression. A second case showed a focus of ++ staining in BA18 (which would correspond to Braak stage V), but otherwise was typically grade II, which was the grade assigned. A third case showed a slightly unusual pericapillary granular tau reactivity, but this was not neuritic and did not affect staging.

### Other Age-related Tau pathologies

**Fig. 1:**
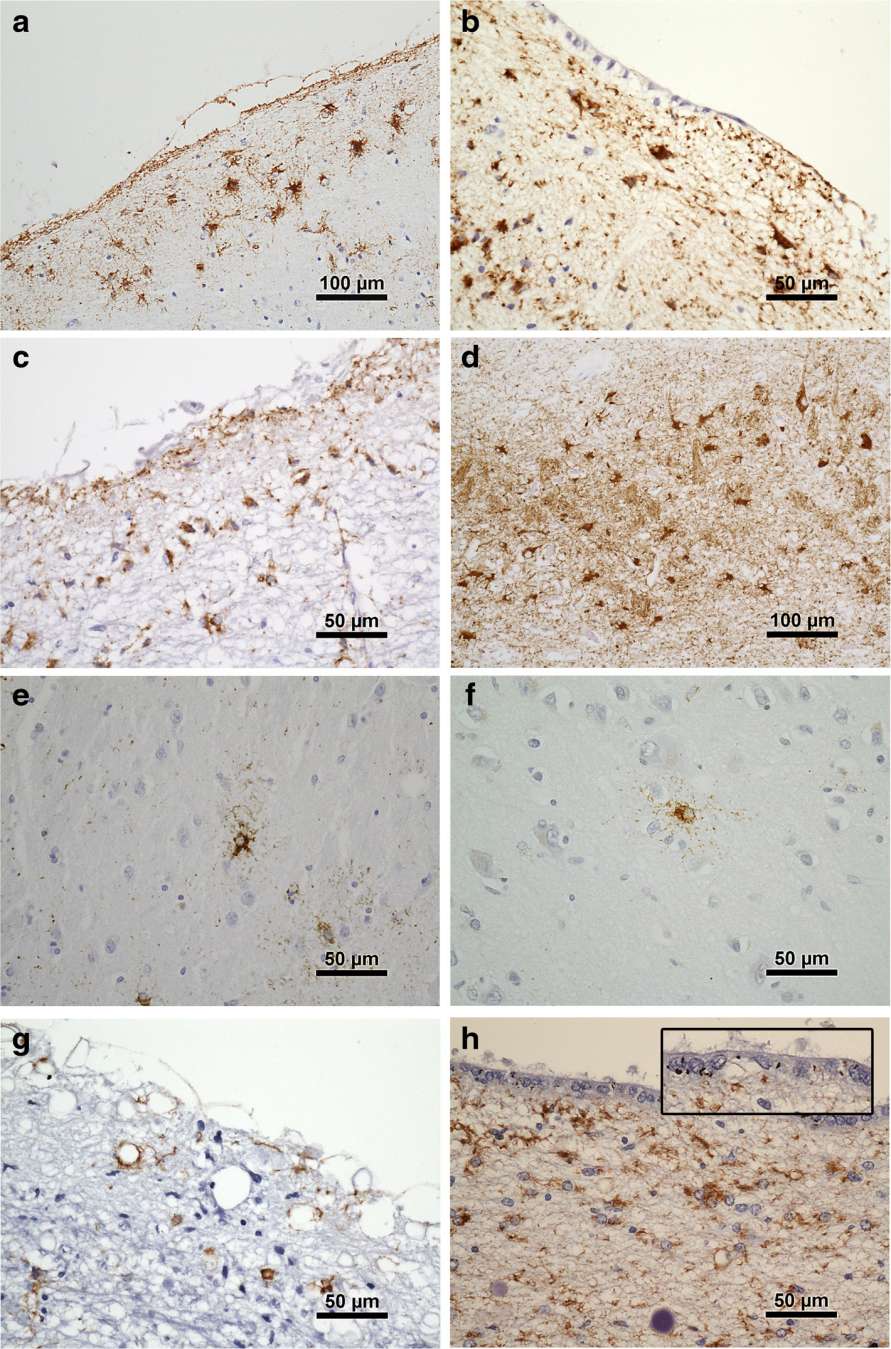
Examples of tau pathologies in the cohort. **a**. Subpial TSA temporal cortex. Note layer of subpial tau + neurites (arrow). **b**. Subependymal TSA hippocampal region. **c**. Midbrain subpial TSA and neurites. **d**. TSA in CA2 region of hippocampus. **e**. Astrocyte with finely tau immunoreactive processes in striatum and, **f**. in temporal cortex. **g**. Tau + neurites pons, without TSA. **h**. Tau positive neurites in subependymal and ependymal (arrow and inset) location associated with TSA, hippocampal region

**Fig. 2:**
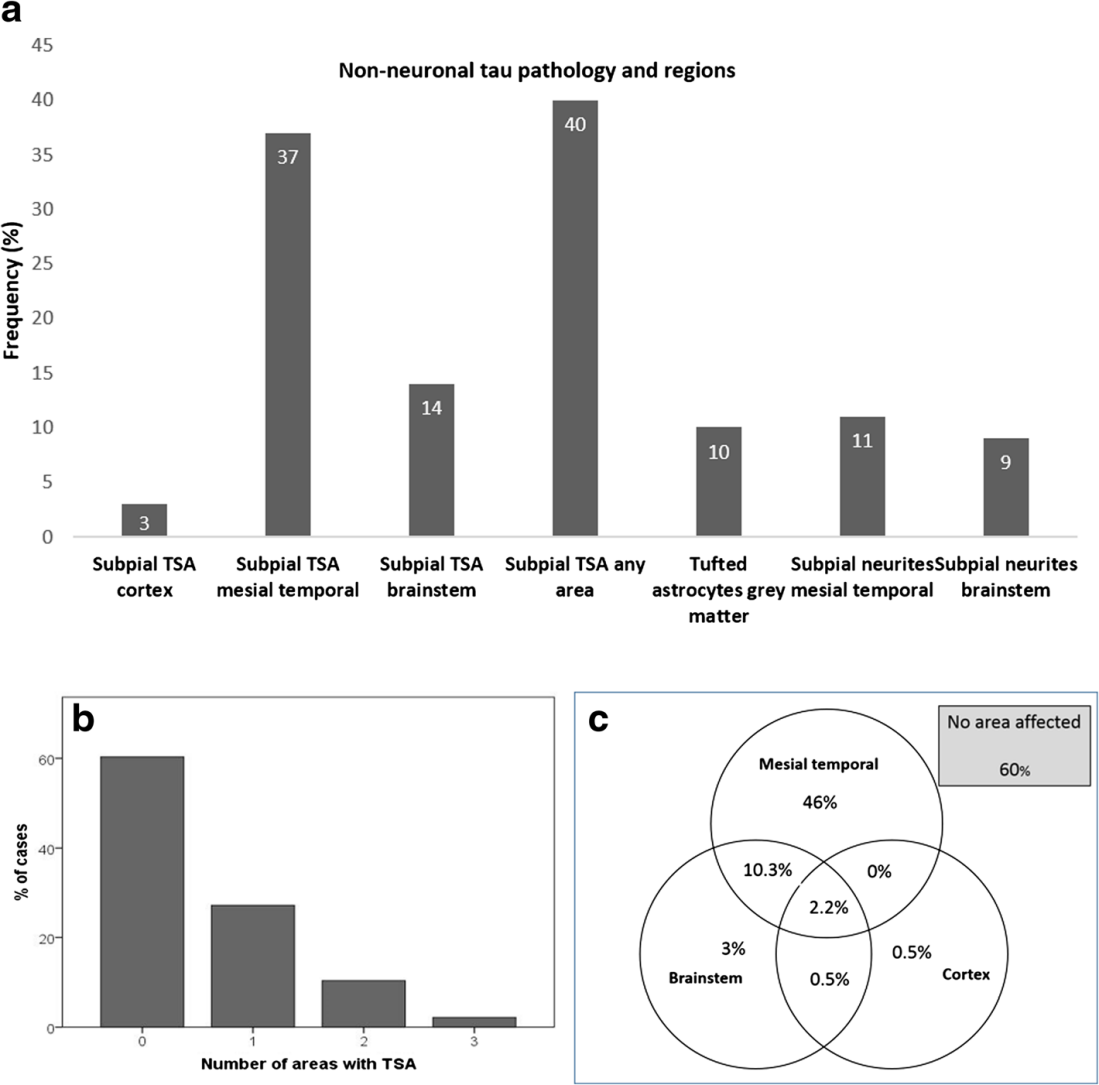
**a**. Frequencies of glial and subpial neuritic tau pathologies. **b**. Distribution of number of areas involved by TSA. **c**. Venn diagram showing the distribution of areas affected with TSA

Tau pathology in astrocytes was of varying morphology and was common in the cohort (Fig. 1). TSA were common in subpial or subependymal regions (40 % in any area examined) (Fig. 2). They were present in one of these regions in 27 % of cases, in two regions in 10 % and in three regions in 2 % and were most frequent in the order mesial temporal region, brainstem (midbrain or pons), cortical regions. They were also observed in cortical white matter in two cases. Tau-positive astrocytes in grey matter were TSA or tufted astrocytes, or astrocytes with a similar morphology, varying from ramified profiles to astrocytes with finely granular tau-immunoreactive processes [15]. Tufted astrocytes (including those with finely granular processes) were observed in 18 cases (10 %) and could be seen in cortical, mesial temporal or subcortical grey matter, usually as isolated or sparse positive cells. There were significantly more areas with TSA in brains with tufted astrocytes than in those without [0.95(0.22) vs 0.50(0.06), t(181) = −2.45, 95 % CI(mean difference) = 0.39; 0.61, *p* = 0.016].

Tau-positive neuritic processes were observed in the immediate subpial area, often associated with corpora amylacea and TSA, and also in subependymal areas, or even within the ependyma (Fig. 1). Subpial neuritic processes showed significant associations with TSA in the mesial temporal [*X*^2^(1) = 61.3, *p* < 0.001) and brainstem (*X*^2^(1) = 47.9, *p* < 0.001] areas. Subpial neurites were observed in the mesial temporal region in 68 (37 %) of cases and in the brainstem regions in 60 (32 %) of cases. Argyrophilic grains, identified on tau immunohistochemistry, were observed in 9 (5 %) of cases.

### Relationship between BNE NT stage, and Braak NFT stage, and TSA

There was a strong correlation between BNE NT stage (BrainNet Europe) and Braak NFT stage [*r* = 0.84, *p* < 0.001, Table 1]. There were weak correlations between TSA and Braak NFT stage [*r* = 0.17, *p* = 0.023, Table 2] and between TSA and BNE NT stage [*r* = 0.17, *p* = 0.023, Table 3]; the identical correlation values here reflects the similarity in BNE NT stage and Braak NFT stage and the use of a non-parametric test.

**Table 1:** Distribution of BNE NT stage and braak NFT stage

Braak NFT stage	BNE NT stage *n*(cell %)					
	I	II	III	IV	V	VI
0	5(2.7)	0(0.0)	0(0.0)	0(0.0)	0(0.0)	0(0.0)
I	9(4.9)	3(1.6)	0(0.0)	0(0.0)	0(0.0)	0(0.0)
II	20(10.9)	18(9.8)	8(4.4)	1(0.6)	0(0.0)	0(0.0)
III	3(1.6)	18(9.8)	11(6.0)	2(1.1)	1(0.6)	1(0.6)
IV	0(0.0)	3(1.6)	26(14.2)	14(7.7)	3(1.6)	0(0.0)
V	0(0.0)	0(0.0)	2(1.1)	7(3.8)	6(3.3)	7(3.8)
VI	0(0.0)	0(0.0)	0(0.0)	1(0.6)	3(1.6)	11(6.0)

**Table 2:** Distribution of TSA according to braak NFT stage

Braak NFT stage	TSA *n*(cell %)			
	None	1 area	2 areas	3 areas
0	5(2.7)	0(0.0)	0(0.0)	0(0.0)
I	11(6.0)	1(0.6)	0(0.0)	0(0.0)
II	30(16.4)	13(7.1)	4(2.2)	0(0.0)
III	21(11.5)	10(5.5)	3(1.6)	2(1.1)
IV	21(11.5)	16(8.7)	8(4.4)	1(0.6)
V	10(5.5)	8(4.4)	4(2.2)	0(0.0)
VI	12(6.6)	2(1.1)	0(0.0)	1(0.6)

**Table 3:** Distribution of TSA according to BNE NT stage

BNE NT stage	TSA *n*(cell %)			
	None	1 area	2 areas	3 areas
I	32(17.5)	4(2.2)	1(0.6)	0(0.0)
II	24(13.1)	12(6.6)	5(2.7)	1(0.6)
III	20(10.9)	19(10.4)	7(3.8)	1(0.6)
IV	15(8.2)	6(3.3)	3(1.6)	1(0.6)
V	7(3.8)	4(2.2)	2(1.1)	0(0.0)
VI	12(6.6)	5(2.7)	1(0.6)	1(0.6)

#### Relationship between BNE NT stage, braak NFT stage and TSA with age according to dementia status

For the further analyses presented, Braak NFT stage was grouped as 0/II, III/IV V/BNE NT stage as I/II, III/IV V/VI and TSA as none, 1 area and 2/3 areas. Their distribution according to dementia status is shown in Table 4. The relationships between Braak NFT stage, BNE NT stage and TSA with age, according to dementia status, were verified using ordinal logistic regression with BNE NT stage, Braak NFT stage and TSA stage as dependent variables and age as independent variable (Table 5). The relationships between BNE NT stage, Braak NFT stage with age were significant only among participants without dementia. The relationship between TSA and age was not significant in either group, but TSA did show a trend to increase with age among participants with dementia, and also when the demented and non-demented groups were considered together, though this was marginal with a low odds ratio [OR 1.06; 95 % CI (OR) 1.02;1.11, *p* = 0.003].

**Table 4:** Distribution of Braak NFT stage, BNE NT stage and TSA according to dementia status

	No dementia	Dementia	Unknown
Braak NFT Stage			
0/II	36(56.3)	23(35.9)	5(7.8)
III/IV	31(37.8)	48(58.5)	3(3.7)
V/VI	2(5.4)	35(94.6)	0(0.0)
BNE NT Stage			
I/II	43(54.4)	32(40.5)	4(5.1)
III/IV	24(33.3)	44(61.1)	4(5.6)
V/VI	2(6.3)	30(93.8)	0(0.0)
TSA			
None	16(32.0)	31(62.0)	3(6.0)
1 area	4(17.4)	19(82.6)	0(0.0)
2/3 areas	49(44.6)	56(50.9)	5(4.6)

**Table 5:** Ordinal logistic regression analyses investigating relationships of Braak NFT stage, BNE NT stage and TSA with age according to dementia status

Dependent	No dementia			Dementia		
variables	OR	95 % CI(OR)	*p*	OR	95 % CI(OR)	*p*
Braak NFT St.	1.08^a^	(1.01; 1.15)	0.017	0.99	(0.94; 1.04)	0.674
BNE NT St.	1.09^a^	(1.02; 1.16)	0.014	0.98	(0.93; 1.03)	0.473
TSA	1.02	(0.96; 1.09)	0.504	1.06	(1.00; 1.12)	0.072

^a^Explanatory note: OR here means that that for each extra year of life: the chances of moving from braak 0/II to III/IV (or braak III/IV to V/VI) increases in 1.08 times and the chances of moving from NTS I/II to III/IV (or NTS III/IV to V/VI) increases in 1.09 times

#### Risk prediction of dementia based on age, BNE NT stage, braak NFT stage and TSA

To verify the added value of investigating Braak NFT stage, BNE NT stage and TSA on age in terms of dementia risk prediction we compared a logistic regression model where age was the only predictor of dementia with models where Braak NFT stage, BNE NT stage and TSA were added with age as independent variables (Table 6). We verified the difference in models by testing the equality of areas under the ROC curves (AUC) based on the predicted values coffered by those models. The AUC in the models that had age and Braak NFT stage [*X*^2^(1) = 10.1, *p* = 0.002) or BNE NT stage (*X*^2^(1) = 9.7, *p* = 0.002] were predictors was significantly larger (12 and 11 % larger respectively) than the model that just had age as a predictor. However, adding TSA to age did not significantly increase the AUC [*X*^2^(1) = 0.77, *p* = 0.381]. There was no significant difference between the model that included Braak NFT stage with the model that included BNE NT stage in terms of prediction of dementia [*X*^2^(1) = 0.15, *p* = 0.699] (Table 6).

**Table 6:** Logistic regression analyses investigating relationships between NTS, braak NFTS and TSA

	OR	95 % CI(OR)	*p*	AUC	95 % CI(AUC)
Age, years	1.11	(1.06; 1.17)	<0.001	0.71	(0.63; 0.79)
Braak NFT St^a^				0.80	(0.73; 0.86)
III/IV	1.60	(0.75; 3.42)	0.226		
V/VI	23.01	(4.90; 108.00)	<0.001		
BNE NT St^b^				0.79	(0.72; 0.86)
III/IV	1.90	(0.92; 3.92)	0.082		
V/VI	17.43	(3.78; 80.26)	<0.001		
TSA^c^				0.72	(0.64; 0.80)
2 areas	1.47	(0.69; 3.13)	0.313		
2/3 areas	3.03	(0.91; 10.08)	0.070		

^a^Reference: Braak NFT Stage 0-II. ^b^Reference: BNE NT Stage I/II. ^c^Reference TSA 0/1 area

## Discussion and conclusions

Tau pathology associated with brain ageing appears to form a continuum with that associated with AD. There is a hierarchy of progression that differs from that of amyloid deposition, as defined in staging schemes such as that of Thal et al. [34]. Indeed, NFT formation may precede Aβ-amyloid deposition, with occasional NFTs appearing at quite young ages [6]. Tau aggregation in mesial temporal structures without significant Aβ encountered in older brains has given rise to the term “primary age-related tauopathy” (PART) [7], but this may reflect early tau deposition preceding Aβ-stages, and thus be part of the ageing-AD spectrum [8]. The lack of a discontinuity between age-related tauopathy and the tauopathy described in AD is consistent with subclinical tau and amyloid representing early stages of AD, with increasing likelihood of dementia with increasing burdens of pathology. It is therefore important to understand the different forms of tau pathology in brain ageing, their pathogenesis and relationship to dementia.

Much of the work on tau pathology and pathogenesis has focused on the neurofibrillary tangle, located within the neuronal soma. However, the NFT pathology seen in AD, some tauopathies, and ageing is accompanied by tau pathology within the neuritic compartment in the form of NTs. Given that tau modification affects its ability to regulate microtubule function [26], neuritic-compartment tau pathology may be an important contributor to dysfunction of axons and dendrites, and failure of synaptic support. The contribution of neuritic tauopathy to cognitive function is therefore an important question. The strong correlation observed in this population-representative cohort between BNE NT stage and Braak NFT stage suggests that NT formation in general parallels that of NFT formation, and supports the objective of the BrainNet Europe approach to the simplification of the staging process. The fact that both NTs and NFTs are so overlapped may suggest that their development is synchronous, but our data, which is cross-sectional, do not allow us to determine this definitively. The relationship of tau to dementia is thus to neuronal tau in general, not specifically to either NFTs or NTs. It would be further of interest to determine if NTs and NFTs are occurring within the same populations of neurones. Like NFT staging, we found that NT progression is highly stereotypic so that only ~1 % of cases deviated from the BNE NT staging scheme, and this was due to isolated foci of neocortical NT formation. The BNE NT staging scheme therefore appears to be reliable and relatively easy to apply.

BNE NT stage and Braak NFT stage both increase with age among the participants without dementia, consistent with the known age effects on prevalence of tau pathology over the age-course [6], but this age-effect was not seen within the individuals who had dementia, who had a tendency to be at the higher stages. This age-effect in non-demented individuals was not seen with TSA.

Using a logistic regression model, inclusion of BNE NT stage adds additional information about the dementia status compared with age alone. Although BNE NT stage assigns some cases to a different stage compared with Braak NFT protocol (Table 1), both schemes have a similar effect in dementia models, so that the addition of BNE NT stage does not provide additional information beyond Braak NFT staging. A common assumption in dementia neuropathology regards Braak NFT stages V and VI as being highly predictive of dementia. Comparison of NT and NFT stages show that 10 cases with Braak NFT stages V or VI have a BNE NT stage of IV or less, similarly 5 cases of NT stage V or VI were scored Braak NFT stage IV or less. For either protocol a score of stages V or VI is highly predictive of dementia; only 1 in 20 cases for either protocol would be incorrectly assigned to a dementia diagnosis on this metric. However, as previously reported in the CFAS cohort, between 1 in 3 (BrainNet Europe) and 1 in 4 (Braak) elderly individuals with stage 0-II tauopathy have dementia at death, and approximately 60 % have dementia with stage III-IV pathology using either protocol [22, 25]. Thus, as with NFTs, there is considerable overlap in burdens of NT pathology between those with dementia and those without in this population sample.

A spectrum of astroglial tau-pathology is increasingly recognised as a common feature in brain ageing, distinct from the forms of astrocytic tau, such as astrocytic plaques, tufted astrocytes and globular inclusions that have diagnostic significance for specific tauopathies [15]. There has been limited evaluation of these forms of age-related astroglial tau and a recent consensus paper has suggested a more harmonised approach to its assessment under the umbrella term of ageing-related tau astrogliopathy (ARTAG) [16]. Astroglial tau pathology is common in this aged cohort, most commonly TSA which, as one of the major constituent subtypes of ARTAG, are suggested to be an age-related tauopathy rather than disease-specific. Located particularly in subpial, subependymal and perivascular locations, these have been described in mesial temporal structures especially in ageing brains [19, 29]. Recognised best with AT8-immunohistochemistry, they appear to express 4R-tau and can be variable detected with antibodies to p62. Variable staining with Gallyas silver preparations suggests that some of the tau is fibrillary. Tufted astrocytes, which are a feature of progressive supranuclear palsy, were identified in 10 % of our cases, suggesting that they can be seen in ageing brain. However, they were present as isolated cells, and the morphology of many of these showed more finely granular processes, suggesting that they are more akin to the “astrocytes with finely granular tau immunoreactivity in processes” which have been described in mesial temporal structures in the elderly [15, 17]. We now show that these isolated forms of astrocyte tau pathology are not confined to mesial temporal structures in the ageing brain, whilst their significant association with TSA supports the concept that are part of the constellation of age-related astroglial tauopathy.

We demonstrated that the frequency of TSA increases with ageing in this cohort of older people, among whom more that 75 % were 85 years and over at death. There was, however, only a positive trend with age when participants with dementia and without were considered together, and this had a low odds ratio. Other studies suggest that they are uncommon in younger brains, not included in our study [29]. Thus, whilst age may be an important influence on their pathogenesis, as for neuronal tau, ageing is only a marginal effect within an already aged-population. A fuller study of the relationship to age will require a cohort with a wider ageing spectrum.

Whilst TSA are particularly prevalent in mesial temporal areas, we further show that they develop around the brainstem, which is an important site in the early development of AD pathology [33], and less frequently in neocortical areas. Although not formally assessed in this study, they are also very common in a subpial location beneath the substantia innominata and in the semilunar and ambient gyri. Thus they are particular prevalent at basal surfaces of the brain. These are also areas with early NT and NFT formation. Our previous study, restricted to the mesial temporal area, did not demonstrate a relationship between TSA and Braak NFT stage [19]. However, *this* study, which takes account of three brain areas, does demonstrate weak correlation of TSA with both BNE NT stage and Braak NFT stage, suggesting that TSA formation may be loosely associated with neuronally-derived tau pathology.

TSA were significantly associated with tau in subpial neurites, right at the brain surface. These, along with subependymal tau, are in neurites in close proximity to CSF. Indeed some tau appears to be present within ependyma. The cellular location of this staining remains to be determined, but neuronal processes are described that contact ventricular CSF [35]. It could be hypothesised that this represents a disposal pathway for aggregated tau. The presence of corpora amylacea in this zone might also support this as a disposal route. An alternative possibility is that tau at this location might represent an early site of induction in response to CSF factors (Personal Communication, Udo Rub, Frankfurt, Germany). In either case, the presence of tau at this location is very common and may be important in the pathogenesis of age and AD-associated tau. Astrocytes have been previously shown to take up Aβ-aggregates, contributing to plaque progression and moving these aggregates towards the pial surface. The association of TSA with subpial neuritic tau raises the possibility that they result from astrocytic uptake of aggregated tau along this disposal pathway. However, in situ induction of tau in astrocytes still needs to be formally excluded.

There are a number of limitations to this study. CFAS is a population-based study. This has advantages for this type of analysis, where an unbiased approach is ideal, but it also imposes limitations compared to clinicopathologically defined case–control cohorts, to which the population-based approach is complementary (reviewed in [32]). For example, dementia in CFAS is defined by a dichotomizing, algorithmic approach. This approach is reliable compared to clinical assessment, is ideal for population studies, and has provided high quality epidemiological data on dementia. [13, 21, 36]. It does, however, lack the more textured approach of detailed neuropsychological assessment that can be employed in well-defined clinic-derived populations, thereby limiting assessment of the relationship of grading schemes to specific aspects of cognitive impairment in CFAS.

The areas chosen were primarily to assess the BNE NT stage, as defined by the BNE protocol, but this may constitute limited sampling for comprehensive assessment of prevalence of tau cytopathologies, including astroglial tau. This is particularly relevant for TSA, which can be focal and which are also seen in subpial basal forebrain areas not assessed in this sampling protocol. Thus TSA prevalence may be underestimated. Semi-quantification of TSA in this study was also limited to presence in one, two or three areas. Assessment of further areas and more quantitative approaches may produce a more graded scaling, allowing more powerful exploration of relationships. Sampling guidelines currently being suggested are likely to have a valuable impact here [16]. Whilst this is a sizeable cohort, the low odds ratios in some analyses (especially for TSA), suggest that larger numbers may better establish some relationships. Furthermore, the CFAS cohort is an aged cohort; assessment in cohorts with a wider age spectrum are required to better elucidate broader ageing relationships. Lastly, although the AT8 antibody is widely used in research and diagnosis, this is a tau prevalence study based on a single antibody recognising a specific tau modification.
